# Editorial: Plant-Soil Interactions Under Changing Climate

**DOI:** 10.3389/fpls.2020.621235

**Published:** 2020-12-18

**Authors:** Sanna Sevanto, Charlotte Grossiord, Tamir Klein, Sasha Reed

**Affiliations:** ^1^Earth and Environmental Sciences Division, Los Alamos National Laboratory, Los Alamos, NM, United States; ^2^Plant Ecology Research Laboratory, School of Architecture, Civil and Environmental Engineering, Ecole Polytechnique Federale de Lausanne, Lausanne, Switzerland; ^3^Functional Plant Ecology, Community Ecology Unit, Swiss Federal Institute for Forest, Snow, and Landscape Research WSL, Lausanne, Switzerland; ^4^Department of Plant and Environmental Sciences, Weizmann Institute of Science, Rehovot, Israel; ^5^U. S. Geological Survey, Southwest Biological Science Center, Moab, UT, United States

**Keywords:** climate change, greenhouse gas (CH_4_, N_2_O, CO_2_), microbiome, ecosystem, vegetation

The health and well-being of plants and soil is crucial for all life on Earth. It is well-known that vegetation cover follows climatic zones, and plants respond to climatic drivers such as temperature and precipitation (Seddon et al., 2016; Kattge et al., 2020). It is also well-known that plant health depends on the properties and health of the soil (Ephrath et al., 2020), and that strong interactions among biota above and belowground dictate the functioning of both realms (Van der Putten et al., 2013). Yet, soils and the processes occurring belowground are often considered a “black box,” and are treated very simplistically in our efforts to understand, quantify, and model the future of the planet. Our understanding of the interactions between plants and soils is also far from complete and offers some of the most important research frontiers in community ecology, biogeochemistry, and global change science.

This Research Topic gathers contributions to the growing literature highlighting the importance of interactions between plants and soil to their mutual health and productivity, as well as to their contributions to greenhouse gas emissions and the climate system. The soil in itself is a complex system consisting of the mineral soil matrix mixed with organic materials, mostly of plant origin. Organic matter is decomposed, altered, and modified by the soil microbiome, consisting of a myriad of bacteria, fungi, algae, viruses, and archaea. The products of these processes are typically greenhouse gases such as CO_2_, N_2_O, or CH_4_ that are released to the atmosphere, and dissolved organic carbon (DOC) that remains in the soil or is lost through the hydrologic system (Ontl and Schulte, 2012). These processes also transform key nutrients into forms available for plant use (Jacoby et al., 2017). The rate of transformation and products created depend on the type and amount of organic material, the composition of the microbiome, as well as the chemical and physical environment affected by the soil matrix properties, climate, and weather. These controls, in turn, also influence the composition of the soil-, rhizosphere-, and plant-microbiome. In addition to producing organic materials for decomposition through litter, plants add a layer of complexity to the system by exuding relatively simple carbohydrates to feed their preferred microbiome, which helps the plant to thrive through improved access to water and nutrients (Jacoby et al., 2020) and possibly releasing plant growth-promoting chemical signals (van Dam and Bouwmeester, 2016). The complexity of plant-soil interactions, and the lack of effective methods to analyze microbiome composition and function until recent years, mean that the field is open for discoveries. Recent technical developments are revolutionizing the field across multiple scales and for numerous components of the plant-soil system, including exposing the dynamics of interactions, identifying differences in microbial communities and how the environment influences both (Sergaki et al., 2018).

This Research Topic includes both experimental and review studies addressing many of the key aspects of plant-soil interactions, using novel approaches and innovative perspectives. Soil, plants, and microorganisms form a triangle of six possible interactions; all of which are represented in the diverse studies in this collection. The atmosphere—its gas composition and climate—makes for a fourth player, also studied here. Taken as a whole, readers will find updated information on burning questions such as (1) how plants and the environment influence soil microbial communities (Deng J. et al.; Gehring et al.; Karlowsky et al.; Liu et al.; Mandrubia et al.; Na et al.); (2) how soil and the rhizosphere microbiome affect the function of plants (Egamberdieva et al.; Ulrich et al.; Vargas et al.); (3) how plant-microbiome interactions influence nutrient availability and soil chemistry (Salmon et al.; Wei et al.); and (4) how plants, soil, and the microbiome influence greenhouse gas emissions (Bréchet et al.; Deng N. et al.).

This collection of studies covers a wide variety of environments from agricultural systems (Egamberdieva et al.; Na et al.), to temperate forests (Deng J. et al.; Deng N. et al.; Gehring et al.; Liu et al.; Ulrich et al.), subalpine forests (Wei et al.) tropical forests (Bréchet et al.), grasslands (Karlowsky et al.; Vargas et al.; Wei et al.), and the Arctic (Salmon et al.). The Topic also includes a study addressing the effects of plant invasions and range expansion across latitudes on the associated soil microbiome (Mandrubia et al.). The work collated here covers a variety of spatial and temporal scales, as well as the effects of different abiotic stressors or recovery from disturbance on the soil and soil microbiome. The variety of topics highlights the state of the art in the field. The complexity and remaining unknowns of the field do not yet allow for final overarching conclusions. Nevertheless, similar to studies in any rapidly growing field, each contribution adds important knowledge to the catalog of information that forms the basis for building theories and conceptual models for understanding the function of the system. Harnessing microbiomes to improve soil health, control greenhouse gas emissions, and improve plant stress tolerance and performance has a huge potential for resolving some of the largest challenges of humanity from controlling and mitigating climate and environmental change to ensuring food security.

We hope that you enjoy this collection of studies and allow it to inspire your research and quest for understanding how plants and soils interact; how they influence the world around us; and how the changing world impacts them.

## Author Contributions

SS drafted the story line of the editorial. CG, TK, and SR refined the story line and contributed references and insights to the impact of the papers included in this research topic.

## Conflict of Interest

The authors declare that the research was conducted in the absence of any commercial or financial relationships that could be construed as a potential conflict of interest.

## References

[B1] Ephrath J. E. Klein T. Sharp R. E. Lazarovitch N. (2020). Exposing the hidden half: root research at the forefront of science. Plant Soil 447, 1–5. 10.1007/s11104-019-04417-y

[B2] Jacoby R. Peikert M. Succurro A. Koprivova A. Kopriva S. (2017). The role of soil microorganisms in plant mineral nutrition –current knowledge and future directions. Front. Plant Sci. 8:1617. 10.3389/fpls.2017.01617 28974956 PMC5610682

[B3] Jacoby R. P. Chen L. Schwier M. Koprivova A. Kopriva S. (2020). Recent advances in the role of plant metabolites in shaping the root microbiome. F1000Res. 9:F1000 Faculty Rev-151. 10.12688/f1000research.21796.1 32148778 PMC7047909

[B4] Kattge J. Bonisch G Diaz S. Lavorel S. Prentice I. C. Leadley P.. (2020). TRY plant trait database –enhanced coverage and open access. Global Change Biol. 26, 5202–5216. 10.1111/gcb.15212 31891233

[B5] Ontl T. A. Schulte L. A. (2012). Soil carbon storage. Nat. Educ. Knowl. 3:35. Available online at: https://www.nature.com/scitable/knowledge/library/soil-carbon-storage-84223790/

[B6] Seddon A. W. R. Macias-Fauria M. Long P. R. Benz D. Willis K. J. (2016). Sensitivity of global terrestrial ecosystems to climate variabilit*y. Natu*re 531, 229–232. 10.1038/nature16986 26886790

[B7] Sergaki C. Lagunas B. Lidbury I. Gifford M. L. Schafer P. (2018). Challenges and approaches in microbiome research: from fundamental to applied. Front. Plant Sci. 9:1205. 10.3389/fpls.2018.01205 30174681 PMC6107787

[B8] van Dam N. M. Bouwmeester H. J. (2016). Metabolomics in the rhizosphere: tapping into belowground chemical communication. Trends Plant Sci. 21, 256–265. 10.1016/j.tplants.2016.01.008 26832948

[B9] Van der Putten W. H. Bardgett R. D. Bever J. D. Bezemer T. M. Casper B. B. Fukami T. (2013). Plant–soil feedbacks: the past, the present and future challenges. J. Ecol. 101, 265–276. 10.1111/1365-2745.12054

